# Supplementary figures and images for: Coherent chaos in a recurrent neural network with structured connectivity
Source: PLoS Comput Biol. 2018 Dec 13;14(12):e1006309. doi: 10.1371/journal.pcbi.1006309 (PMC6307850; doi:10.1371/journal.pcbi.1006309)

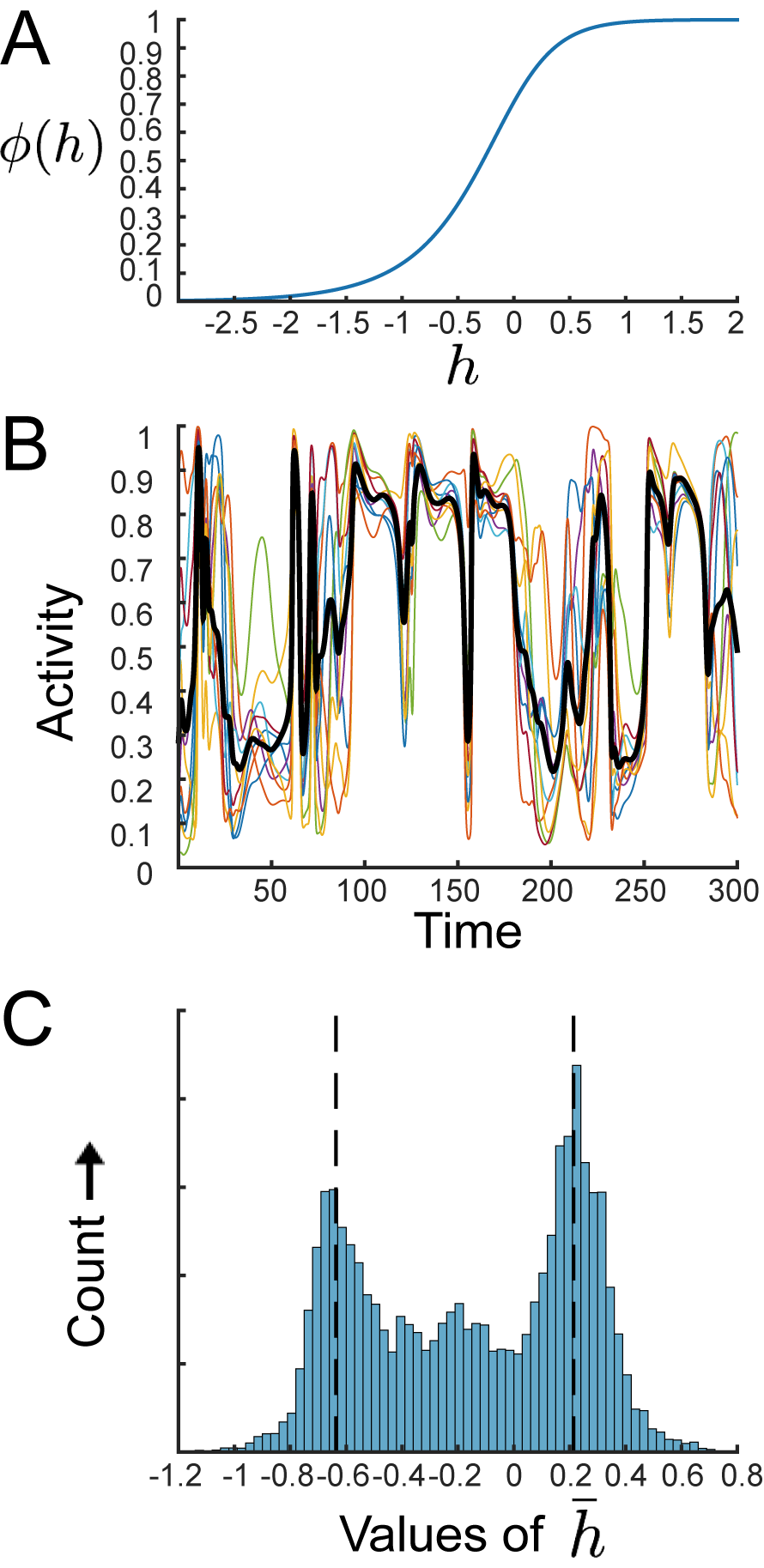

Supplement: S1 Fig — (A) We use a non-symmetric transfer function ϕ(h) = (1 + exp − βh)−p with β = 4 and p=12. (B) Activity trace of coherent activity ϕ¯(t) in black and 10 randomly chosen neurons ϕi(t) displays coherent switching between slow states. (C) Histogram of values of coherent current, h¯, displays bimodality with peaks near the critical values predicted by theory where ϕ′(h¯)=1g. Simulations for N = 2000 and g = 2 with row balance. (TIF) [file pcbi.1006309.s001.tif]

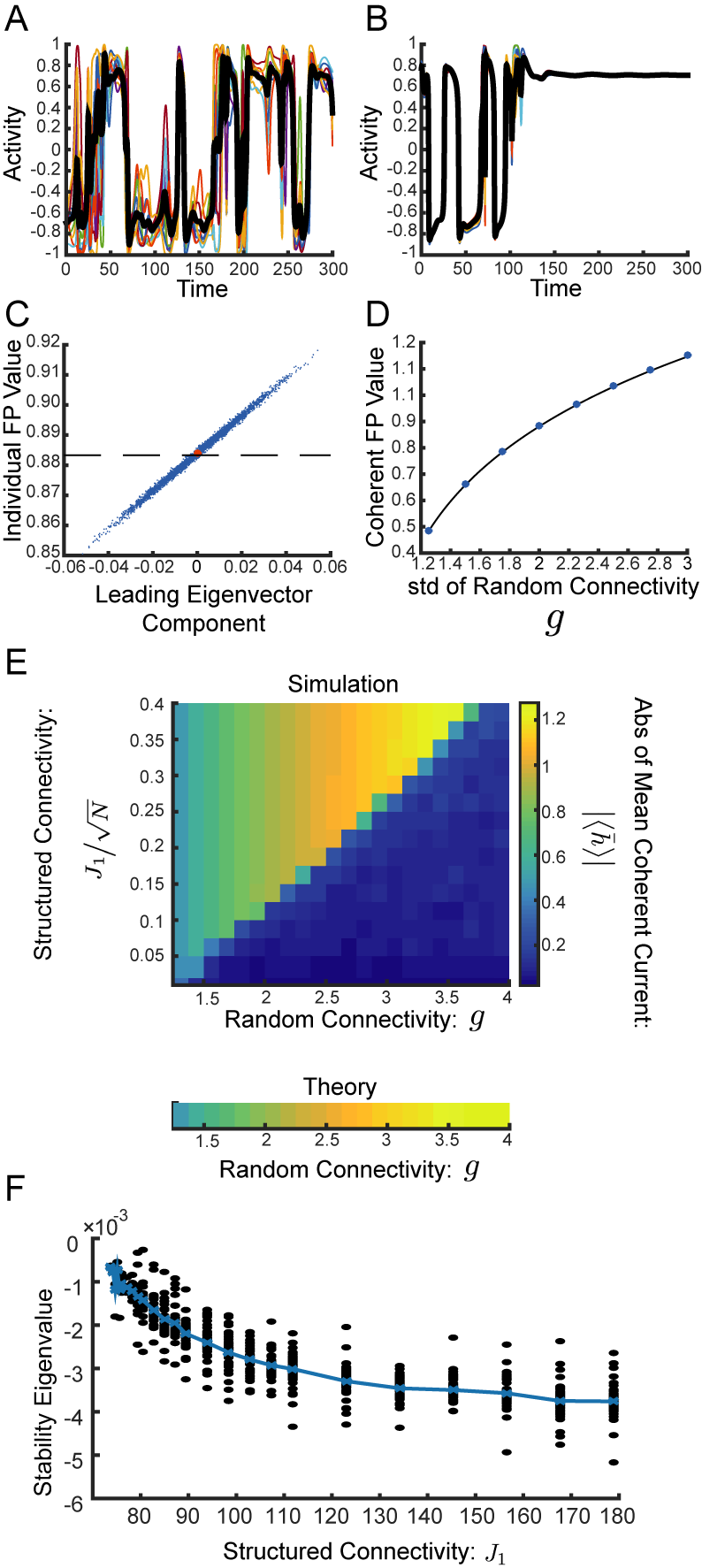

Supplement: S2 Fig — (A) Sample chaotic dynamics for J1 = 6.32. (B) Sample dynamics of same connectivity realization as in (A) but with J1 = 63.2. (C) Scatterplot of all hi* at fixed point, plotted against individual components of the leading eigenvector, ui1. Red dot is value of coherent mode at FP. Black dashed line is FP value predicted from theory. (D) Value of coherent mode at FP, h¯*, as a function of the standard deviation of the random connectivity, g. Black line is prediction from theory: h¯c=sech-1(1g). (E) Phase diagram for a single realization with real leading eigenvalue. Colormap shows the absolute value of the mean coherent current over a single trial, ∣〈h¯〉∣ which is close to zero when the network is chaotic and non-zero when at a fixed point. The bar below shows the fixed point value predicted by theory, h¯c, which is independent of J1. (F) Stability eigenvalue at fixed point, i.e. leading eigenvalue of the Jacobian, -1+J˜ijϕ′(hj*), as a function of J1 for a specific realization of the random connectivity. The fixed point exhibits marginal stability independent of J1. Networks in all panels have row balance. N = 4000. (TIF) [file pcbi.1006309.s002.tif]

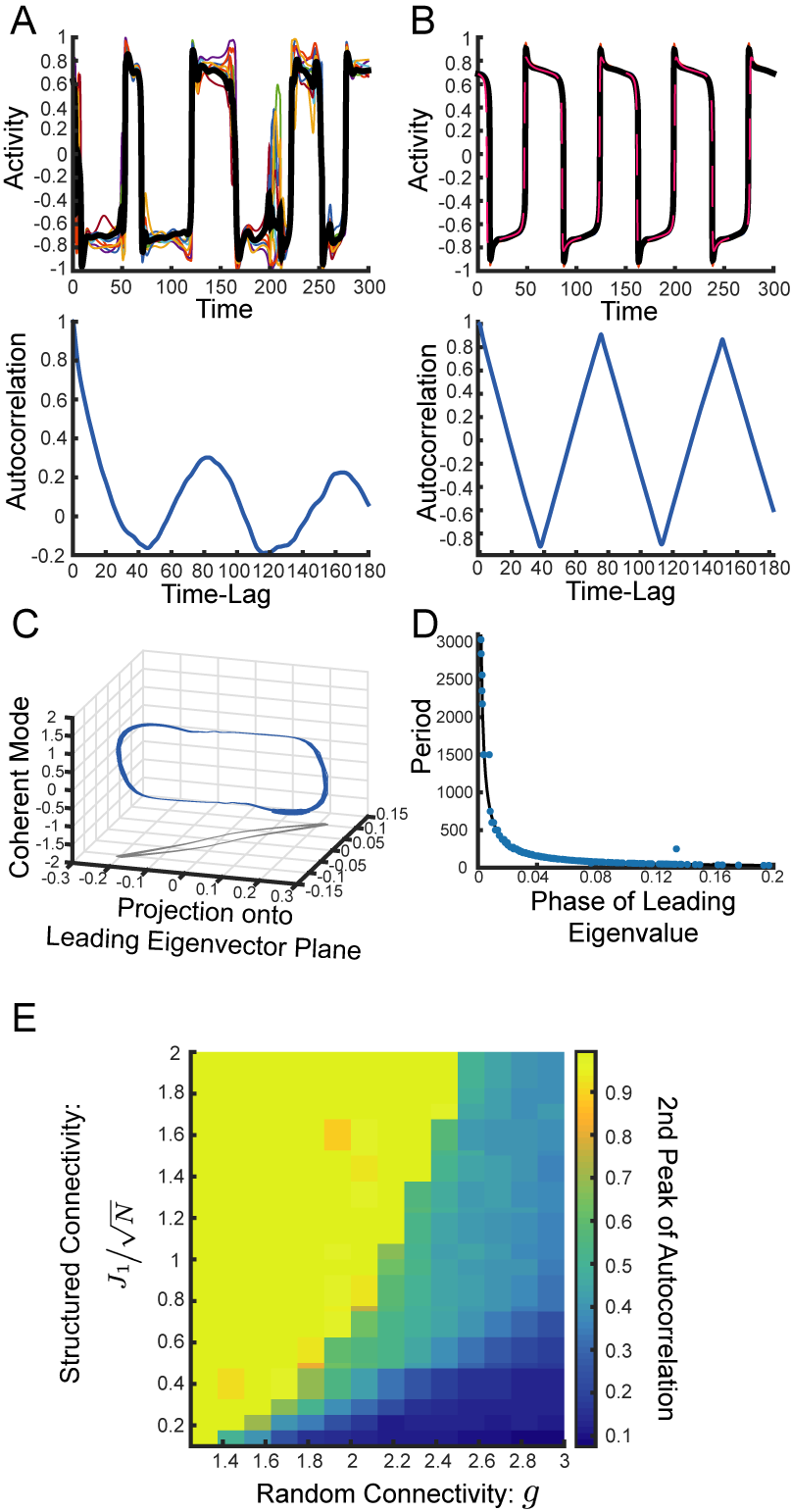

Supplement: S3 Fig — (A) Top: Sample chaotic dynamics for J1 = 15.8. Bottom: Autocorrelation of coherent mode shows oscillatory ringing. (B) Same connectivity realization as (A) but with J1 = 126. Dashed pink line in top panel is prediction from solving the three-dimensional dynamics. Autocorrelation shows near-perfect oscillations. (C) Projection of the full dynamics of (B) onto coherent mode and the real and imaginary parts of the leading eigenvector. These three dimensions account for more than 0.99 of the total variance of the dynamics. Gray projection onto the leading eigenvector plane accounts for 0.98 of the variance of the residual currents. (D) Scatterplot of period of oscillations plotted against the phase of the leading eigenvalue, Imλ1Reλ1, of J^, for 219 different realizations of the random connectivity. Black line shows prediction from theory, T=2πReλ1Imλ1. (E) Phase diagram for a single connectivity realization with complex leading eigenvalue. Colormap shows the second peak of the normalized autocorrelation of the coherent mode. Networks in all panels have row balance. N = 4000. (TIF) [file pcbi.1006309.s003.tif]

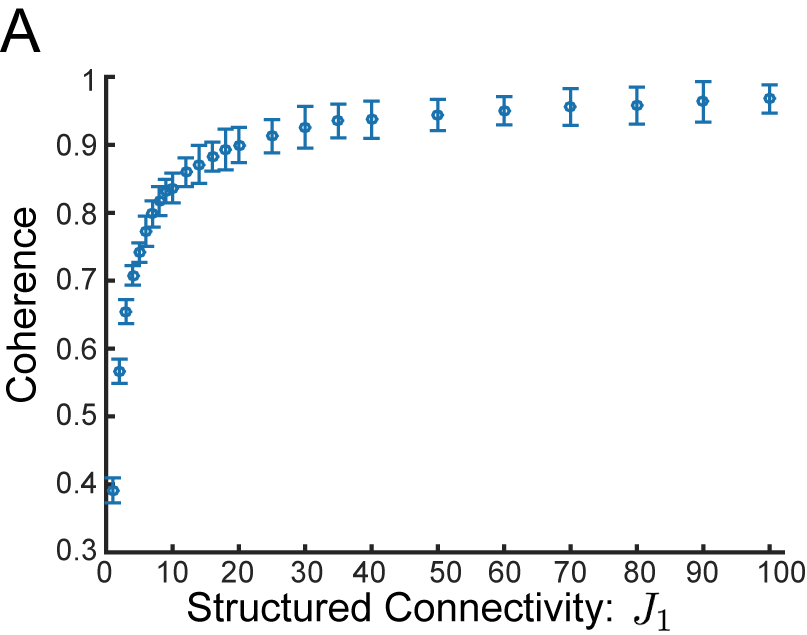

Supplement: S4 Fig — (A) Plot of coherence, χ, vs strength of structured connectivity, J1, for networks of size N = 16000 with row balance. Dots display average over realizations, bars display standard deviation. Only chaotic realizations included (those not found to be at a fixed point or a limit cycle—see Methods). More than 20 realizations per value of J1. For J1 = 100, 22 out of 30 realizations were chaotic and the average coherence among these realizations was 0.963. (TIF) [file pcbi.1006309.s004.tif]
