# Supplementary material for: Coherent chaos in a recurrent neural network with structured connectivity
Source: PLoS Comput Biol. 2018 Dec 13;14(12):e1006309. doi: 10.1371/journal.pcbi.1006309 (PMC6307850; doi:10.1371/journal.pcbi.1006309)
Supplement: S1 Appendix — We analyze the realization-dependence of the network with and without row balance, derive the perturbative dynamic mean-field equations, and derive the non-chaotic solutions to the dynamics in the limit of strong structured connectivity. (PDF) [file pcbi.1006309.s005.pdf]

Coherent chaos in a recurrent neural network with structured connectivity – Appendix

Itamar Daniel Landau[1] and Haim Sompolinsky[1,2]

[1]Edmond and Lily Safra Center for Brain Sciences, The Hebrew University of Jerusalem, Jerusalem, Israel

[2]Center for Brain Science, Harvard University, Cambridge, MA, USA

Contents

1 Exact Decomposed Dynamics and Row Balance **2**

2 Perturbative Dynamic Mean-Field Theory in the Limit of Weak Structured Connectivity **4**

3 Analysis of the Limit of Strong Structured Connectivity with Row Balance **6**

1 Exact Decomposed Dynamics and Row Balance

We write the full dynamics without row balance:

$$\frac{\mathrm{d}\mathbf{h}}{\mathrm{d}t} = -\mathbf{h} + \mathbf{J}\boldsymbol{\phi} + \frac{J_1}{\sqrt{N}}\boldsymbol{\xi}\boldsymbol{\nu}^T\boldsymbol{\phi} \tag{1}$$

and we define

$$\bar{h} \equiv \frac{1}{N}\boldsymbol{\xi}^T\mathbf{h} \quad \bar{\phi} \equiv \frac{1}{N}\boldsymbol{\xi}^T\boldsymbol{\phi} \tag{2}$$

$$\delta\mathbf{h} \equiv \mathbf{h} - \bar{h}\boldsymbol{\xi} \quad \delta\boldsymbol{\phi} = \boldsymbol{\phi} - \bar{\phi}\boldsymbol{\xi} \tag{3}$$

Applying these definitions to the full dynamics (and noting that $\boldsymbol{\nu}^T\boldsymbol{\phi} = \boldsymbol{\nu}^T\delta\boldsymbol{\phi}$), the exact coherent mode dynamics are

$$\frac{\mathrm{d}\bar{h}}{\mathrm{d}t} = -\bar{h} + \frac{J_1}{\sqrt{N}}\boldsymbol{\nu}^T\delta\boldsymbol{\phi} + \frac{\boldsymbol{\xi}^T}{N}\mathbf{J}\boldsymbol{\phi} \tag{4}$$

and by subtracting these from the full dynamics of $\mathbf{h}$, the decomposed dynamics are:

$$\frac{\mathrm{d}\delta\mathbf{h}}{\mathrm{d}t} = -\delta\mathbf{h} + \mathbf{J}\boldsymbol{\phi} - \frac{\boldsymbol{\xi}\boldsymbol{\xi}^T}{N}\mathbf{J}\boldsymbol{\phi} = -\delta\mathbf{h} + \hat{\mathbf{J}}\boldsymbol{\phi} \tag{5}$$

where $\hat{\mathbf{J}} \equiv \left(\mathbf{I} - \frac{\boldsymbol{\xi}\boldsymbol{\xi}^T}{N}\right)\mathbf{J}$ as introduced in the main text in Eqns (5) and (6).

We observe that the constraint $\boldsymbol{\xi}^T\delta\mathbf{h} = 0$ must be satisfied automatically by the residual dynamics (Eqn 5), and this can be confirmed by verifying that

$$\frac{\mathrm{d}\left(\boldsymbol{\xi}^T\delta\mathbf{h}\right)}{\mathrm{d}t} = -\left(\boldsymbol{\xi}^T\delta\mathbf{h}\right) \tag{6}$$

In the regime where $J_1 \ll 1$ the ϕ_j are nearly uncorrelated and therefore $\frac{\boldsymbol{\xi}^T}{N}\mathbf{J}\boldsymbol{\phi} \sim O\left(\frac{1}{\sqrt{N}}\right)$ and can be ignored. This yields the approximate coherent dynamics presented in Eqn (4) of the main text.

In the regime with strong structured connectivity we must consider this term in Eqn 4. To that end we write $\boldsymbol{\phi} = \bar{\phi}\boldsymbol{\xi} + \delta\boldsymbol{\phi}$ and also write the transformation of the input mode via the random matrix as

$$\mathbf{J}\boldsymbol{\xi} = a_{\parallel}\boldsymbol{\xi} + \boldsymbol{\xi}^{\perp} \tag{7}$$

where $a_{\parallel}$ is a realization-dependent scalar and $\boldsymbol{\xi}^{\perp}$ is a realization-dependent vector orthogonal to $\boldsymbol{\xi}$. That yields

coherent mode dynamics

$$\frac{\mathrm{d}\bar{h}}{\mathrm{d}t} = -\bar{h} + \frac{J_1}{\sqrt{N}}\boldsymbol{\nu}^T\delta\boldsymbol{\phi} + a_{\parallel}\bar{\phi} + \frac{\boldsymbol{\xi}^T}{N}\mathbf{J}\delta\boldsymbol{\phi} \tag{8}$$

and residual dynamics

$$\frac{\mathrm{d}\delta\mathbf{h}}{\mathrm{d}t} = -\delta\mathbf{h} + \hat{\mathbf{J}}\delta\boldsymbol{\phi} + \bar{\phi}\boldsymbol{\xi}^{\perp} \tag{9}$$

As J_1 increases and the fluctuations in the coherent activity, $\bar{\phi}(t)$, drive feedback in two ways. First of all, $\mathbf{J}$ maps the coherent activity back along the input mode driving direct feedback to the coherent current $\bar{h}$ via the term $a_{\parallel}\bar{\phi}$.

Secondly, $\mathbf{J}$ maps the coherent activity in a realization-dependent direction, $\boldsymbol{\xi}^{\perp}$, orthogonal to the input mode. This drives the residual activity fluctuations $\delta\mathbf{h}$ via the term $\bar{\phi}\boldsymbol{\xi}^{\perp}$, and this biasing of the residual fluctuations may in turn generate feedback to the coherent current through the output mode via $\boldsymbol{\nu}^T\delta\boldsymbol{\phi}$.

Both of these feedback terms are realization dependent, and both of them are canceled via the row balance subtraction

$$\tilde{\mathbf{J}} \equiv \mathbf{J} - \mathbf{J}\frac{\boldsymbol{\xi}\boldsymbol{\xi}^T}{N} \tag{10}$$

which yields exact coherent mode dynamics

$$\frac{\mathrm{d}\bar{h}}{\mathrm{d}t} = -\bar{h} + \frac{J_1}{\sqrt{N}}\boldsymbol{\nu}^T\delta\boldsymbol{\phi} + \frac{\boldsymbol{\xi}^T}{N}\mathbf{J}\delta\boldsymbol{\phi} \tag{11}$$

and residual dynamics

$$\frac{\mathrm{d}\delta\mathbf{h}}{\mathrm{d}t} = -\delta\mathbf{h} + \hat{\mathbf{J}}\delta\boldsymbol{\phi} \tag{12}$$

And in this case the residual dynamics are again uncorrelated so that $\frac{\boldsymbol{\xi}^T}{N}\mathbf{J}\delta\boldsymbol{\phi} \sim O\left(\frac{1}{\sqrt{N}}\right)$ and can again be ignored in the coherent mode dynamics.

Note that $\bar{\phi}$ no longer drives feedback to either the residual or the coherent dynamics. Nevertheless the dynamics are still coupled in both directions as $\delta\boldsymbol{\phi}$ depends on $\bar{h}$.

2 Perturbative Dynamic Mean-Field Theory in the Limit of Weak Structured Connectivity

We derive the dynamic mean-field equations in the limit of small J_1 using a perturbative approach. We write the mean-field dynamics of the residuals as

$$\frac{\mathrm{d}\delta h_i}{\mathrm{d}t} = -\delta h_i + \eta_i \tag{13}$$

and the coherent component as

$$\frac{\mathrm{d}\bar{h}}{\mathrm{d}t} = -\bar{h} + J_1 m \tag{14}$$

where η_i and m are assumed to be uncorrelated, mean-zero Gaussians. For general J_1 the assumption of Gaussianity fails, therefore we assume $J_1 \ll 1$.

The autocorrelation of η_i is given by

$$\left[\langle \eta_i(t)\, \eta_i(t+\tau)\rangle\right] = \left[\sum_{j,k} J_{ij} J_{ik} \langle \phi_j(t)\, \phi_k(t+\tau)\rangle\right] \tag{15}$$

where we have introduced [] as the notation for averaging over realizations. We assume that ϕ_j is independent of J_{ij} and so the terms $j \neq k$ have average zero over realizations and we get

$$\left[\langle \eta_i(t)\, \eta_i(t+\tau)\rangle\right] = g^2 C(\tau) \tag{16}$$

where

$$C(\tau) \equiv \left[\langle \phi_j(t)\, \phi_j(t+\tau)\rangle\right] \tag{17}$$

The autocorrelation of m is given by

$$\left[\langle m(t)\, m(t+\tau)\rangle\right] = \frac{J_1^2}{N}\left[\sum_{j,k} \nu_j \nu_k \phi_j(t)\, \phi(t+\tau)\right] \tag{18}$$

And again the $j \neq k$ terms fall in the realization average so that

$$\left[\langle m(t)\, m(t+\tau)\rangle\right] = J_1^2 C(\tau) \tag{19}$$

Next we define the autocorrelation of the residuals

$$\Delta_{\delta}\left(\tau\right) \equiv \left[\left\langle \delta h_i\left(t\right)\delta h_i\left(t+\tau\right)\right\rangle\right] \tag{20}$$

and the autocorrelation of the coherent current

$$\bar{\Delta}\left(\tau\right) \equiv \left[\left\langle \bar{h}\left(t\right)\bar{h}\left(t+\tau\right)\right\rangle\right] \tag{21}$$

and we can follow previous work [3, 2] and write the dynamic mean-field equations for $\Delta_{\delta}\left(\tau\right)$ as

$$\left(1-\frac{\partial^2}{\partial\tau^2}\right)\Delta_{\delta}\left(\tau\right) = g^2 C\left(\tau\right) \tag{22}$$

and for $\bar{\Delta}\left(\tau\right)$ as

$$\left(1-\frac{\partial^2}{\partial\tau^2}\right)\bar{\Delta}\left(\tau\right) = J_1^2 C\left(\tau\right) \tag{23}$$

Next we note that for $J_1 \ll g$ we assume that $\left|\bar{h}\right| \ll 1$ so we have $\phi_i = \phi\left(\delta h_i + \xi_i\bar{h}\right) \approx \phi\left(\delta h_i\right) + \xi_i\phi'\left(\delta h_i\right)\bar{h}$. Therefore we have that to leading order

$$C\left(\tau\right) \approx \left[\left\langle \phi\left(\delta h_i\left(t\right)\right)\phi\left(\delta h_i\left(t+\tau\right)\right)\right\rangle\right] \tag{24}$$

and then following previous results [3, 2] we can write this to leading order as an integral over Gaussians:

$$C\left(\tau\right) \approx \int_{-\infty}^{\infty} Dz \left(\int_{-\infty}^{\infty} Dx\,\phi\left(\sqrt{\Delta_{\delta}\left(0\right)-\Delta_{\delta}\left(\tau\right)}x + \sqrt{\Delta_{\delta}\left(\tau\right)}z\right)\right)^2 \tag{25}$$

Note that it is possible to compute the sub-leading correction term as well, but for our purposes this is unnecessary. We suffice it to observe that to leading order, the self-consistency equation for $\Delta_{\delta}\left(\tau\right)$ (Eqn 22) reduces to the identical equation for that of a random network without structured component ($J_1 = 0$)[3], and that $\bar{\Delta}\left(\tau\right)$ contributes only to the sub-leading correction. Following [3, 2] then Eqn 22 can be solved yielding $\Delta_{\delta}\left(\tau\right) \approx \Delta_0\left(\tau\right)$, where $\Delta_0\left(\tau\right)$ is the autocorrelation when $J_1 = 0$.

The dynamic equation for $\bar{\Delta}\left(\tau\right)$ is identical to that for $\Delta_{\delta}\left(\tau\right)$ except with J_1 in place of g, so we conclude (as presented in Eqn (9) of the main text) that the resulting leading order autocorrelation of the coherent mode is

$$\bar{\Delta}\left(\tau\right) \approx \frac{J_1^2}{g^2}\Delta_0\left(\tau\right) \tag{26}$$

Thus for $J_1 \ll g$ fluctuations in the coherent input are driven passively by the random source which is generated self-consistently by the residual fluctuations, and the resulting autocorrelation of the coherent mode is simply a scaled version of the autocorrelation of the residuals.

It is worth noting that for $J_1 \sim g$ the assumption of Gaussianity is broken due to the cross-correlations between the ϕ_j.

3 Analysis of the Limit of Strong Structured Connectivity with Row Balance

In the limit of large J_1 we assume $\delta h_i \ll 1$, and approximate $\phi_j \approx \phi\left(\xi_j \bar{h}\right) + \phi'\left(\bar{h}\right)\delta h_j$, where we have made use of the symmetry of the transfer function and the binary restriction on ξ_j. Note that this linearization clearly holds without symmetric transfer function for the case of uniform $\xi_j = 1$ as well.

Using the random connectivity with row balance constraint, $\tilde{\mathbf{J}}$, and following the exact decomposition above (Eqns 5 and 4) this yields dynamical equations:

$$\frac{\mathrm{d}\delta\mathbf{h}}{\mathrm{d}t} = -\delta\mathbf{h} + \phi'\left(\bar{h}\right)\hat{\mathbf{J}}\delta\mathbf{h} \tag{27}$$

$$\frac{\mathrm{d}\bar{h}}{\mathrm{d}t} = -\bar{h} + \frac{J_1}{\sqrt{N}}\phi'\left(\bar{h}\right)\boldsymbol{\nu}^T\delta\mathbf{h} + \phi'\left(\bar{h}\right)\frac{\boldsymbol{\xi}^T}{N}\mathbf{J}\delta\mathbf{h} \tag{28}$$

In this regime $\bar{h}$ acts as a dynamic gain on the local synaptic currents through $\phi'\left(\bar{h}\right)$. Given $\bar{h}$ the equation for the residual currents is linear and therefore their dynamics can be decomposed in the eigenbasis of the matrix

$$\hat{\mathbf{J}} \equiv \mathbf{P}_\xi \mathbf{J} \tag{29}$$

where $\mathbf{P}_\xi = \mathbf{I} - \frac{\boldsymbol{\xi}\boldsymbol{\xi}^T}{N}$ is the projection matrix onto the the subspace orthogonal to $\boldsymbol{\xi}$.

We observe a fine-point not noted in [1]: It may seem intuitive that the eigenvalues of $\tilde{\mathbf{J}}$ determine the dynamics. In fact, as we show these eigenvalues are identical to those of $\hat{\mathbf{J}}$. However, had we ignored the constraint $\boldsymbol{\xi}^T\delta\mathbf{h} = 0$ then the residual dynamics would have been determined by $\mathbf{J}$ and its eigenvalues, and these are not the same as those of $\hat{\mathbf{J}}$.

We claim that $\hat{\mathbf{J}} = \mathbf{P}_\xi \mathbf{J}$ and $\tilde{\mathbf{J}} = \mathbf{J}\mathbf{P}_\xi$ have the same eigenvalues. Suppose λ is an eigenvalue of $\hat{\mathbf{J}}$ with associated eigenvector $\mathbf{u}$, then $\mathbf{u}$ must be orthogonal to $\boldsymbol{\xi}$. If $\mathbf{Ju}$ is orthogonal to $\boldsymbol{\xi}$ as well, then $\tilde{\mathbf{J}}\mathbf{u} = \hat{\mathbf{J}}\mathbf{u} = \lambda\mathbf{u}$, and we are done. Otherwise we can write $\mathbf{Ju} = \lambda\mathbf{u} + a\boldsymbol{\xi}$, and thus $\tilde{\mathbf{J}}\left(\lambda\mathbf{u} + a\boldsymbol{\xi}\right) = \mathbf{J}\lambda\mathbf{u} = \lambda\left(\lambda\mathbf{u} + a\boldsymbol{\xi}\right)$ so that λ is also an eigenvalue of $\tilde{\mathbf{J}}$. Suppose now that λ is an eigenvalue of $\tilde{\mathbf{J}}$. Again if the associated eigenvector is orthogonal to $\boldsymbol{\xi}$ then it is also an eigenvector of $\hat{\mathbf{J}}$ with eigenvalue λ and we are done. Otherwise we write the eigenvector of $\tilde{\mathbf{J}}$ as $\mathbf{u} + a\boldsymbol{\xi}$ and then we have $\tilde{\mathbf{J}}\left(\mathbf{u} + a\boldsymbol{\xi}\right) = \mathbf{Ju} = \lambda\left(\mathbf{u} + a\boldsymbol{\xi}\right)$. Therefore $\hat{\mathbf{J}}\mathbf{u} = \lambda\mathbf{u}$.

We write the eigenvectors as $\mathbf{u}^{(i)}$ with $\hat{\mathbf{J}}\mathbf{u}^{(i)} = \lambda_i \mathbf{u}^{(i)}$. We write the vector of residual current as $\delta\mathbf{h} = \sum_i c_i \mathbf{u}^{(i)}$ and note that as mentioned above $\mathbf{u}^{(i)} \perp \boldsymbol{\xi}$, so that the constraint $\boldsymbol{\xi}^T \delta\mathbf{h} = 0$ is satisfied. This yields dynamics

$$\frac{\mathrm{d}c_i}{\mathrm{d}t} = \left(-1 + \phi'\left(\bar{h}\right)\lambda_i\right)c_i \tag{30}$$

The only (marginally) stable, non-zero fixed point is achieved with $c_1 \neq 0$ and $c_i = 0$ for all $i > 1$. And the fixed-point equation is

$$c_1^*\left(1 - \phi'\left(\bar{h}\right)\lambda_1\right) = 0 \tag{31}$$

This fixed point only exists if λ_1 is real, and yields a fixed-point requirement for $\bar{h}^*$:

$$\bar{h}^* = \phi'^{-1}\left(\frac{1}{\lambda_1}\right) \approx \phi'^{-1}\left(\frac{1}{g}\right) \tag{32}$$

In order to close the loop we turn to the fixed point equation for the coherent dynamics. Ignoring the term $\phi'\left(\bar{h}\right)\frac{\boldsymbol{\xi}^T}{N}\mathbf{J}\delta\mathbf{h}$, which yields an $O\left(\frac{1}{\sqrt{N}}\right)$ correction we find:

$$\bar{h}^* = \frac{J_1}{\sqrt{N}}\phi'\left(\bar{h}\right)\boldsymbol{\nu}^T\delta\mathbf{h}^* \tag{33}$$

which in turn, using $\delta\mathbf{h}^* = c_1\mathbf{u}^{(1)}$, yields a solution to leading order for c_1: $c_1^* = \frac{\sqrt{N}\bar{h}^*\lambda_1}{J_1\boldsymbol{\nu}^T\mathbf{u}^{(1)}}$, as reported in [1].

If λ_1 is complex there is no fixed point but rather a limit-cycle solution to the dynamics of the complex-valued c_1 exists with $\delta\mathbf{h}\left(t\right) = \mathrm{Re}\left[c_1\left(t\right)\mathbf{u}^{(1)}\right]$, and $c_i = 0$ for all other eigenmodes. Assuming $\bar{h}\left(t\right)$ is periodic with period T, we can separate variables and integrate Eqn 30 in order to find $c_1\left(t\right)$ is given by

$$c_1\left(t\right) = c_1\left(0\right)\exp\left(-t + \lambda_1\Phi\left(t\right)\right) \tag{34}$$

for $t \leq T$, where $\Phi(t) = \int_0^t \mathrm{d}s\, \phi'\left(\bar{h}(s)\right)$. Writing $c_1(0) = \left|c_1^0\right| \exp(i\theta_0)$ and using $\mathrm{Re}\lambda_1 \approx g$, this gives

$$c_1(t) = \left|c_1^0\right| \exp\left(-t + g\Phi(t)\right) \exp\left(i\left(\theta_0 + \mathrm{Im}\lambda_1 \Phi(t)\right)\right) \tag{35}$$

A limit cycle in phase with $\bar{h}(t)$ means $c_1(T) = c_1(0)$ and this requires that both $g\Phi(T) = T$ and also $\mathrm{Im}[\lambda_1]\,\Phi(T) = 2\pi$. From the first requirement we find that the average value of ϕ' over a period must be the critical value:

$$\left\langle \phi'\left(\bar{h}\right) \right\rangle = \frac{\Phi(T)}{T} = \frac{1}{g}. \tag{36}$$

And combining the second requirement yields an expression for the period (Eqn **??**, as reported in [1] as well):

$$T = 2\pi \frac{\mathrm{Re}\lambda_1}{\mathrm{Im}\lambda_1} \tag{37}$$

We can further write a self-consistency expression for $\bar{h}(t)$ by taking $c_1(t)$ as given by Eqn 35 and integrate over the coherent mode dynamics:

$$\frac{\mathrm{d}\bar{h}}{\mathrm{d}t} = -\bar{h} + \mathrm{Re}\left[\frac{J_1}{\sqrt{N}} \boldsymbol{\nu}^T \mathbf{u}^{(1)} c_1(0)\, \phi'\left(\bar{h}(t)\right) \exp\left(-t + \lambda_1 \Phi(t)\right)\right] \tag{38}$$

which yields

$$\bar{h}(t) = \bar{h}(0) \exp(-t) + \mathrm{Re}\left[\frac{J_1}{\sqrt{N}} \frac{\boldsymbol{\nu}^T \mathbf{u}^{(1)}}{\lambda_1} c_1(0) \exp\left(-t + \lambda_1 \Phi(t)\right)\right] \tag{39}$$

Without loss of generality we assume that $\bar{h}(0) = \bar{h}^c = \phi'^{-1}\left(\frac{1}{g}\right)$, then

$$\bar{h}^c \approx c_1^0 \frac{J_1 \left|\boldsymbol{\nu}^T \mathbf{u}^{(1)}\right|}{\sqrt{N} g} \cos\left(\theta_0 + \mathrm{Im}\left(\boldsymbol{\nu}^T \mathbf{u}^{(1)}\right)\right) \tag{40}$$

This is analogous to the fixed point equation for $\bar{h}^*$ and c_1^*. In both cases the requirement that $\delta h_i = c_1 u_i^{(1)} \ll 1$ requires that c_1 be maximally $O(1)$ and motivates our conjectures about the realization-dependence and system-size scaling of the transition out of chaos. In particular, we expect and confirm numerically that the critical value of J_1 for transition to either fixed point or limit cycle is inversely proportional to $\left|\boldsymbol{\nu}^T \mathbf{u}^{(1)}\right|$ and grows with network size (see main text and Fig 7).

In the case of complex leading eigenvalue, simulations confirm that a projection of the full synaptic current dynamics into the coherent mode and the leading eigenvector plane (consisting of real and imaginary parts of $\mathbf{u}^{(1)}$) accounts for well over 0.99 of the total variance. Even restricting ourselves to the variance of the residuals, δh_i, we find that 0.98 of the variance is restricted to the leading eigenvector plane (Fig S3).

For $N = 4000$ we simulate 219 realizations of random connectivity with complex leading eigenvalue and find that for sufficiently large J_1 all but one of these realizations yield highly oscillatory dynamics with period predicted nearly perfectly by theory (Fig S3).

We note that in the limit of large N we expect that the typical size of the imaginary component of the leading eigenvalue, λ_1, shrinks such that the typical period grows. These longer period oscillations are characterized by square-wave-like shape in which the dynamics of the coherent component slows around the critical value $\bar{h}^c = \phi^{-1}\left(\frac{1}{\mathrm{Re}\lambda_1}\right)$, which is identical to the fixed-point value of $\bar{h}$ when λ_1 is real. (Fig S3)

The fraction of realizations with real leading eigenvalue in the large N limit has not been calculated analytically to our knowledge. We find numerically that this fraction appears to saturate roughly around $\frac{3}{10}$ for $N \gtrsim 8000$.

References

[1] Luis Carlos García Del Molino, Khashayar Pakdaman, Jonathan Touboul, and Gilles Wainrib. Synchronization in random balanced networks. *Physical Review E - Statistical, Nonlinear, and Soft Matter Physics*, 88(4):1–5, 2013.

[2] Jonathan Kadmon and Haim Sompolinsky. Transition to chaos in random neuronal networks. *Physical Review X*, 5(4):1–28, 2015.

[3] Haim Sompolinsky, A. Crisanti, and H. J. Sommers. Chaos in random neural networks. *Physical Review Letters*, 61(3):259–262, 1988.
